# Prescribing Cascade as a Therapeutic Error: A Danger for Geriatric Patients with Multimorbidity

**DOI:** 10.3390/geriatrics11020037

**Published:** 2026-03-31

**Authors:** Adrian Bryła, Jarosław Woroń, Miłosz Miedziaszczyk, Barbara Lorkowska-Zawicka, Beata Bujak-Giżycka, Daniel Orzechowski, Paulina Połetek, Wojciech Pałys

**Affiliations:** 1 Department of Clinical Pharmacology, Chair of Pharmacology, Faculty of Medicine, Jagiellonian University Medical College, Grzegorzecka 16, 31-531 Krakow, Poland; 2 Clinical Pharmacy Team, Ludwik Rydygier Specialist Hospital, Os. Zlotej Jesieni 1, 31-826 Krakow, Poland; 3 Clinical Department of Anesthesiology and Intensive Care and Clinical Pharmacology Consulting Office, University Hospital in Krakow, Macieja Jakubowskiego 2, 30-688 Krakow, Poland; 4 University Center for Monitoring and Research on Adverse Drug Reactions, Sniadeckich 10, 31-531 Krakow, Poland; 5 Pharma Consult, Pharmacotherapy Safety Team, Oberconiowka 30/8, 34-500 Zakopane, Poland; 6 Department of Clinical Pharmacy and Biopharmacy, Poznan University of Medical Sciences, Fredry 26/28, 61-927 Poznan, Poland; 7 Department of Anesthesiology and Intensive Care, SPZOZ Hospital in Brzozów, Bielawskiego 18, 36-200 Brzozow, Poland

**Keywords:** prescribing cascade, polypharmacy, chronic kidney disease, drug safety

## Abstract

The aging of the population and the increasing prevalence of multimorbidity contribute to the widespread use of polypharmacotherapy, which in turn elevates the risk of adverse drug reactions and clinically significant drug–drug interactions. One of the key yet frequently underestimated issues in clinical practice is the prescribing cascade, which occurs when an adverse drug reaction is misinterpreted as a new medical condition, leading to the initiation of an additional medication. This phenomenon is particularly relevant in the older population, in whom altered pharmacokinetics and pharmacodynamics, together with reduced organ reserve, increase susceptibility to adverse drug events, including nephrotoxicity (renal impairment is used throughout the review as a clinically relevant example of organ-specific harm resulting from prescribing cascades, rather than as the sole focus of the analysis). This article discusses the mechanisms and clinical consequences of the prescribing cascade—with particular emphasis on renal function deterioration—as well as strategies for its prevention in the geriatric population. Analysis of the literature indicates that prescribing cascades remain insufficiently recognized in clinical practice, despite the availability of pharmacotherapy assessment tools such as The American Geriatrics Society (AGS) Beers Criteria and the STOPP/START criteria. Documented prescribing cascades have been shown to contribute to deterioration in health status and quality of life, an increased frequency of hospitalizations, and a greater burden on healthcare systems. Particularly concerning are cascades involving cardiovascular, neurological, and analgesic medications, which may induce or exacerbate renal injury, ultimately leading to chronic kidney disease and organ failure. Prescribing cascades represent a significant yet frequently underestimated threat to the efficacy and safety of pharmacotherapy in older adults. Their consequences may extend beyond reduced quality of life and increased treatment costs to include serious complications such as the development of renal failure. Enhancing clinicians’ awareness, conducting systematic medication reviews, and employing validated assessment tools are essential for the identification and prevention of prescribing cascades, thereby reducing the risk of renal injury and improving clinical outcomes.

## 1. Introduction

Population aging and the increasing prevalence of multimorbidity have led to a growing reliance on polypharmacotherapy, which substantially raises the risk of adverse drug reactions; clinically significant drug–drug interactions are a frequent contributor to these events. Among the key yet still underappreciated challenges in clinical practice is the phenomenon known as the prescribing cascade. This occurs when an adverse drug reaction is misinterpreted as a new medical condition, leading to the initiation of an additional medication rather than modification or discontinuation of the causative drug. Prescribing cascades may be unintentional and inappropriate, but in selected clinical circumstances they can also be intentional and appropriate, provided that the benefit–risk balance is favorable and aligned with patient-centered therapeutic goals [1,2]. This problem is particularly pronounced in geriatric patients, in whom altered pharmacokinetics and pharmacodynamics, reduced organ reserve, and the coexistence of multiple chronic conditions significantly increase the risk of adverse drug events, their accumulation, and drug–drug interactions [2,3]. Of particular concern are cascades involving cardiovascular, analgesic, and anti-inflammatory agents, which may contribute to renal impairment and even the development of chronic kidney disease and renal failure. The consequences of prescribing cascades therefore extend beyond reduced quality of life and increased risk of hospitalization, encompassing serious organ complications that place an additional burden on healthcare systems. Despite the availability of tools supporting the evaluation of pharmacotherapy appropriateness, such as the American Geriatrics Society (AGS) Beers Criteria and STOPP/START criteria, prescribing cascades remain insufficiently recognized and often overlooked in clinical practice. This article presents a clinical discussion of the prescribing cascade in the geriatric population, focusing on its mechanisms, potential consequences for renal function, and practical approaches to prevention.

## 2. Risk Factors for Prescribing Cascades

Identification of risk factors for the occurrence of prescribing cascades is a key element in limiting the prevalence of this phenomenon. It reduces negative health consequences for the patient and enables verification and improvement of treatment standards, particularly in patients with multimorbidity. Preventing and eliminating prescribing cascades is therefore an important step toward improving the quality of care and clinical outcomes [4]. Risk factors for adverse prescribing cascades can be grouped into categories related to the medical status of the patient and those that are “non-medical,” associated with the current ease of access to medications and other medical products. In the first category, we include advanced age, multimorbidity, polypharmacy (primarily as a quantitative concept (concurrent use of multiple medications), and overprescribing, often linked to multispecialist treatment provided by several physicians without mutual verification of ongoing pharmacotherapy. Within this group, particular attention should be paid to the risk of renal injury—both acute kidney injury (AKI) and chronic kidney disease (CKD). Many drugs prescribed concurrently as part of cascades (e.g., nonsteroidal anti-inflammatory drugs, ACE inhibitors, diuretics, and nephrotoxic antibiotics) may exert cumulative renal burden, resulting in progressive renal impairment, especially in geriatric patients [5,6,7,8]. The second group of risk factors includes obtaining prescriptions online without direct physician consultation, easy availability of over-the-counter drugs (OTC), and, consequently, the possibility of self-medication and unnecessary supplementation (pathosupplementation—non-indicated, non-contextual overuse of dietary supplements without integration into overall pharmacotherapy). Clinically relevant examples of OTC driven prescribing cascades include NSAID-related fluid retention or blood pressure elevation leading to the initiation of diuretics or antihypertensive therapy; the use of OTC anticholinergic agents (e.g., first-generation antihistamines) causing cognitive impairment or urinary retention, subsequently treated with cholinesterase inhibitors or α_1_-blockers; as well as chronic use of OTC proton pump inhibitors contributing to electrolyte disturbances or osteoporosis, followed by mineral supplementation or additional pharmacotherapy. Additional contributing factors are the widespread promotion of medications and supplements in pharmacies, mass media advertising (television, internet), and pharmacy-based sales promotions. Table 1 summarizes the risk factors for the occurrence of prescribing cascades [5,6,9].

Prescribing cascades may occur in any patient experiencing health deterioration, but age-related physiological changes naturally predispose to worsening health status. Age is a parameter defining the biological and physiological aspects of aging and is associated with the natural predisposition to age-related diseases and their treatment. In this group, renal impairment is particularly prevalent, increasing susceptibility to adverse drug reactions from medications eliminated renally. Consequently, older adults are more likely to develop acute kidney injury as a result of prescribing cascades, and long-term exposure may even induce chronic kidney disease. Accurate data on the physiology and pathology of aging may help reduce unnecessary polypharmacy. However, clinical trials often exclude or insufficiently represent older adults with multimorbidity, resulting in a lack of evidence-based (EBM) guidelines for combination drug therapy in this population. This leads to suboptimal pharmacotherapeutic choices—frequently involving the use of certain “older” drug classes with narrow therapeutic index or anticholinergic burden. The lack of data derived from clinical research also contributes to insufficient use of modern therapies in geriatric patients, often justified by concerns about poor tolerability due to age. On the other hand, unexpected cascades may occur when new medications are introduced without a thorough analysis of age-related pathophysiological changes. In summary, the underrepresentation of older patients with multimorbidity in clinical trials results in a lack of precise evidence-based pharmacotherapeutic guidelines for this population, fostering the occurrence of prescribing cascades and increasing the risk of renal insufficiency [5,6,7,10]. Verification of risk factors facilitates the recognition of prescribing cascades, yet practical tools to help physicians reliably identify cascades, assess them appropriately, and manage them effectively are still lacking. Polypharmacy, although considered a risk factor for prescribing cascades, is undoubtedly an essential element of good prescribing practice in aging populations. At the same time, the lack of systematic evaluation of renal function during therapy selection further exacerbates the risk of cascades leading to acute kidney injury or progression of chronic kidney disease. The classification of cascades as appropriate or inappropriate (intended/unintended) may evolve over time depending on the clinical status of the patient [7,8,11,12]. Therapeutic regimens, typically designed with a disease- and organ-specific focus, often overlook an interdisciplinary assessment of renal function. Failure to account for this aspect promotes therapeutic decisions that result in additive adverse drug effects and, consequently, increase the risk of prescribing cascades. Thus, a key question arises: Why does the risk of prescribing cascades increase in clinical practice? Table 2 presents cause-and-effect relationships of prescribing cascades, including their association with renal injury progression [5,6,7,8,9,11,12,13].

## 3. Examples of Prescribing Cascades and Their Clinical Significance

Prescribing cascades are a common but often underrecognized phenomenon in clinical practice, particularly among older adults [7]. They occur when an adverse drug reaction is misinterpreted as a new medical condition, leading to the initiation of an additional drug rather than recognition of the underlying cause. In the long term, this process not only increases the risk of inappropriate polypharmacy but also heightens the likelihood of drug-related morbidity, hospitalizations, and healthcare costs [1,7,12]. The tables below summarize clinically important prescribing cascades described in recent consensus statements and systematic reviews. Table 3 and Table 4 present cascades that are particularly relevant in older adults, identified through the *ThinkCascades* tool and the international list of potentially inappropriate prescribing cascades (PIPC) [1,7,12]. Table 4 presents a non-exhaustive overview of additional, less frequently discussed prescribing cascades, organized by physiological system, to illustrate the breadth and complexity of this phenomenon rather than to provide management recommendations.

**Figure 1:**
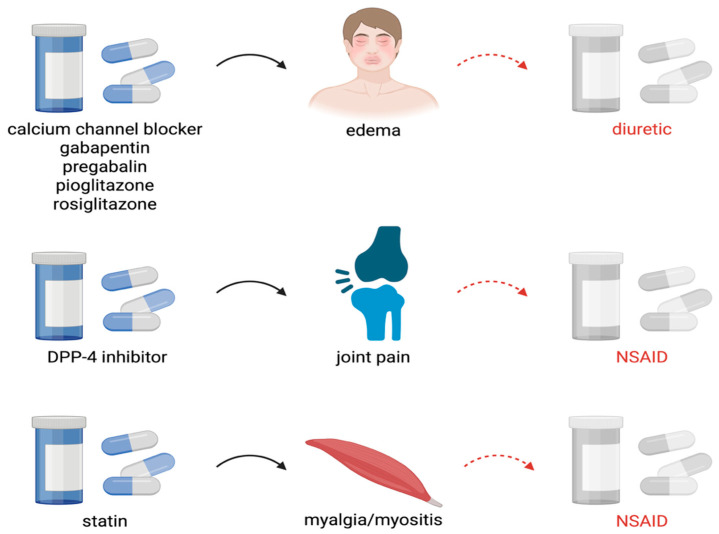
Examples of prescribing cascades affecting kidney function. DPP-4, Dipeptidyl peptidase 4; NSAID, Nonsteroidal anti-inflammatory drug. Created in BioRender. Miedziaszczyk, M. (2025) https://BioRender.com.

The clinical significance of these cascades is multifaceted. First, they frequently contribute directly to deterioration in patients’ functional status. For example, calcium channel blocker-induced peripheral edema treated with diuretics may result in dehydration and falls. Similarly, benzodiazepine-related cognitive impairment treated with cholinesterase inhibitors only adds to the pharmacological burden without addressing the underlying cause. Second, an important yet often underestimated aspect of prescribing cascades is their impact on renal function. The use of diuretics in response to adverse effects of other medications (e.g., edema caused by calcium channel blockers or NSAIDs) may lead to dehydration, electrolyte disturbances, and accelerated decline in glomerular filtration (Figure 1). Consequently, the risk of nephrotoxicity from additional medications, accumulation of drug metabolites, and further progression of kidney disease increases. In older adults, whose renal reserve is already limited, such cascades can significantly increase the risk of acute kidney injury (AKI) or accelerate the development of chronic kidney disease (CKD). Therefore, particular attention should be paid to prescribing cascades involving nephrotoxic agents, drugs altering renal hemodynamics, or medications requiring renal clearance. Third, prescribing cascades often contribute to therapeutic inertia, as clinicians may attribute drug-related symptoms (such as dizziness, hypotension, or worsening renal function tests) to aging or disease progression rather than to adverse drug effects [7,12]. Recent systematic reviews emphasize that prescribing cascades are not random events but rather reproducible and predictable patterns observed across healthcare systems [1]. Recognition of these patterns is therefore critical for implementing preventive strategies such as medication review and deprescribing. In particular, the PIPC list developed by the international expert panel provides clinicians with a practical tool for identifying high-risk cascades, while the *ThinkCascades* tool offers a structured approach to evaluating drug–symptom associations in daily practice [7,12]. Ultimately, the presence of prescribing cascades highlights the importance of regular medication review, interdisciplinary collaboration, and patient education. Raising awareness of well-documented cascades such as those listed above may help clinicians distinguish adverse drug reactions from disease progression—including declining renal function and thereby reduce inappropriate polypharmacy and its associated risks.

## 4. Consequences of Prescribing Cascades

A prescribing cascade can lead to serious health, social, and economic consequences. Although this phenomenon may appear to result from concern for the patient, it often worsens their clinical condition, leads to inappropriate treatment (so-called “therapeutic vicious circles”) and polypharmacotherapy, which turns into polypragmasy. As a result, patients may experience intensified symptoms such as dizziness, drug-induced headaches, falls, delirium, cognitive impairment, balance disorders, orthostatic hypotension, or painsomnia. Importantly, some of these symptoms—such as delirium, balance disturbances, or hypotension—are often mistakenly attributed to the “natural” aging process or progression of the underlying disease, which further delays appropriate intervention. These are only some of the potential consequences of prescribing cascades, which negatively affect patients’ quality of life, leading to reduced mobility and independence and, quite often, the need for hospitalization [12]. Findings from observational studies and systematic reviews, as well as daily clinical practice, clearly indicate that prescribing cascades are a significant risk factor for hospitalization, especially in older adults, where the reason for hospital admission is not the primary disease, but rather adverse drug reactions introduced in response to symptoms caused by previous pharmacotherapy. Examples include falls due to hypnotics or dehydration caused by diuretics prescribed in response to edema induced by NSAIDs or certain antidepressants. In the long term, prescribing cascades also contribute to rising healthcare costs, both direct (such as hospitalizations, diagnostics, and additional specialist consultations) and indirect (loss of independence, the need for continuous care). According to Farrell B and McCarthy LM, deprescribing and early identification of prescribing cascades can significantly reduce systemic expenditures as well as ease the burden on medical staff [7,14]. Economically, prescribing cascades increase resource utilization through higher rates of emergency admissions, prolonged hospital stays, additional laboratory monitoring, and polypharmacy-related adverse drug event management. From a person-centered perspective, they are associated with functional decline, increased frailty, higher fall risk, cognitive impairment, reduced quality of life, and earlier transition to assisted living or long-term care.

## 5. Identification and Prevention of Prescribing Cascades

Currently, there is a lack of practical tools to help physicians and pharmacists reliably detect, correctly assess, and manage prescribing cascades. Ponte et al. developed an 8-point (4 questions) scale to assess the prescribing cascade. Prescribing cascade is assumed to occur if the score is 4 or higher, reflecting the severity of the prescribing cascade. Table 5 presents a tool for assessing the occurrence of drug cascade prescription [15].

The Prescribing Cascade Assessment Tool is based on the framework proposed by Ponte ML et al. The tool was developed to support structured identification of potential prescribing cascades by systematically evaluating whether ADR was misinterpreted as a new clinical condition and treated with an additional medication. Previous applications of this framework demonstrated good face validity and clinical applicability in geriatric pharmacotherapy settings, although it is primarily intended as a clinical reasoning support tool rather than a diagnostic instrument with established psychometric validation.

Dreischulte et al. proposed a tool that assesses whether prescribing cascade is appropriate or inappropriate [6]. The tool is based on the following 6 questions. Although prescribing cascades are most often perceived as problematic, they may be appropriate and therapeutically beneficial in certain clinical situations. McCarthy et al. point out two important issues to consider when deciding on a prescribing cascade. First, consider whether the intentional initiation of a prescribing cascade aligns with the patient’s treatment goals (patient well-being and improved quality of life). Second, consider whether the risks and benefits of the prescribing cascade have been discussed with the patient and whether the patient has been informed of the long-term effects [16]. Table 6 presents the Prescription Cascade Benefit Assessment Tool.

The Prescribing Cascade Benefit Assessment Tool represents a structured clinical decision-support framework designed to evaluate whether continuation of a prescribing cascade may be justified from a benefit–risk perspective. The framework synthesizes principles of deprescribing, adverse drug reaction management, and individualized pharmacotherapy. Similar to the assessment tool described above, it is intended to guide clinical reasoning rather than serve as a formally validated instrument with established psychometric properties.

Analyzing everything that has been described, with particular emphasis on the risk factors for the occurrence of prescribing cascades, as well as clinical practices that increase the risk of their occurrence, we made an attempt to formulate general principles which, when implemented in clinical practice, may help prevent prescribing cascades. Table 7 presents the key points of clinical practice, which we can refer to as a list of good clinical practices [3]. Oligopharmacotherapy refers to a deliberate strategy of limiting pharmacological treatment to medications with clear, evidence-based benefit for the individual patient, in contrast to indiscriminate polypharmacy. Contextual pharmacotherapy emphasizes that prescribing decisions should always be adapted to the patient’s overall clinical context, including multimorbidity, functional status, renal function, life expectancy, and individual therapeutic goals.

Prescribing cascades represent a failure of personalized medicine, as drug-related harms are addressed through additional medications rather than through individualized reassessment of therapy. Deprescribing constitutes a core element of personalized pharmacotherapy, allowing clinicians to reverse therapeutic cascades by aligning treatment with patient-specific factors such as comorbidities, renal function, life expectancy, and treatment goals. From this perspective, prescribing cascades can be understood as therapeutic errors arising from non-contextual, guideline-driven prescribing, whereas deprescribing restores a patient-centered, personalized approach to care.

## 6. Conclusions

Prescribing cascades constitute a clinically significant yet underrecognized problem in geriatric pharmacotherapy. Their occurrence not only promotes inappropriate polypharmacy but also contributes to deterioration in patients’ functional status, an increased risk of hospitalization, and higher healthcare costs. Particularly alarming are cascades involving cardiovascular, analgesic, and neurological agents, which may accelerate the decline of renal function and predispose to acute kidney injury or chronic kidney disease. The findings of this review highlight the urgent need to raise awareness among healthcare professionals about the mechanisms and consequences of prescribing cascades. Regular medication reviews, systematic monitoring of renal function, and the use of validated assessment tools—such as the Beers Criteria, STOPP/START, and PIPC—are essential steps toward minimizing the risk of inappropriate prescribing and preventing renal complications. Interdisciplinary collaboration and patient education further strengthen preventive strategies. Ultimately, addressing prescribing cascades is not only a matter of improving medication safety but also a prerequisite for enhancing the quality of life and clinical outcomes in older adults.

## Figures and Tables

**Table 1:** Risk factors for the occurrence of a prescribing cascade.

Risk Factor	A Mechanism Promoting the Prescribing Cascade	Number of Supporting References
Patient age	Age-related changes in drug metabolism and excretion (pharmacokinetics) as well as tissue responsiveness to drugs (pharmacodynamics) increase susceptibility to adverse drug reactions. Use of high-risk medications in geriatric populations.	≥5
Sex	In certain therapies, women have a higher likelihood of developing prescribing cascades, due to differences in drug absorption rates, hormonal regulation, and metabolic pathways.	2–3
Polypharmacy	A large number of concurrently used drugs increases the probability of drug–drug interactions and adverse events, which may be mistakenly interpreted as new diseases.	≥5
Multimorbidity	The coexistence of multiple comorbidities predisposes to multi-organ dysfunction of varying severity, which affects drug metabolism and elimination. Multiple diseases often require multiple medications.	≥5
Chronic use of multiple medications	Long-term therapies promote the accumulation (“overlapping”) of adverse effects and increase the risk of their misinterpretation.	3–4
Lack of comprehensive medication review	Lack of structured medication review perpetuates the use of unnecessary medications and fosters the development of prescribing cascades. Additionally, the absence of dose reassessment maintains adverse drug effects. Prescribing more drugs instead of minimizing doses, and the failure to consider non-pharmacological alternatives before initiating drug therapy, further contribute to the problem.	3–4
Overprescribing	Insufficient communication about pharmacological interventions implemented by various specialists involved in patient care, including primary care physicians.	2–3
Misinterpretation of symptoms	Adverse drug reactions are misinterpreted as new disease entities, and the patient receives additional medications instead of therapeutic modification.	2–3
Insufficient knowledge of prescribing cascades among healthcare professionals	Insufficient knowledge of the relationship between drugs and symptoms results in a failure to recognize that a new symptom is an adverse effect of a medication rather than a new disease.	2–3
Self-medication	The trend toward a “healthy lifestyle,” widespread availability of OTC drugs, and self-directed treatment, often reinforced by lack of trust in physicians and insufficient pharmacological knowledge.	2–3
Pathosupplementation (unnecessary supplementation without clinical indication)	Dietary supplements as part of alternative treatment approaches. Easy access to various supplements, combined with aggressive marketing, promotes their use. Supplements are often perceived as an element of a “healthy lifestyle” and part of pro-health trends.	2

**Table 2:** Why the risk of prescribing cascades is rising in clinical practice.

Treating the disease rather than the patient with the disease	In the context of appropriately tailored pharmacotherapy, patient characteristics play a crucial role and may constitute significant risk factors for the occurrence of adverse drug reactions, including complex, multifactorial complications arising from the specific nature of the pharmacotherapy employed
Uncritical and context-free application of therapeutic guidelines	A therapeutic guideline must address not only individual diseases, but its appropriate application in a given patient should always be considered within the full spectrum of multimorbidity
Assumption of a class effect within specific drug groups used in the patient	Within specific drug classes, a uniform class effect does not exist, which results from differences in pharmacokinetic and pharmacodynamic (PK/PD) parameters, as well as variability in adverse effect profiles and the associated risk factors for their occurrence.
Fragmentation of multimorbidity in pharmacological decision-making	Decisions regarding the initiation of pharmacotherapy for individual disease entities should always be guided by consideration of the overall spectrum of multimorbidity in the patient
Lack of benefit–risk assessment prior to initiation of pharmacotherapy	Before initiating any medication, the benefit–risk balance must be assessed, as it is influenced by patient-specific characteristics, coexisting risk factors for adverse events, concomitant pharmacotherapy, the use of complementary or alternative medicines, and dietary supplements. Equally relevant is the consumption of broadly defined recreational substances, as well as a history of prior drug-induced adverse reactions
Self-medication and supplementation	They may trigger drug interactions and complications, ultimately modifying the benefit–risk balance of the ongoing pharmacotherapy
Lack of awareness of the adverse effect profiles of prescribed drugs	In the context of polypharmacotherapy, clinicians must have a thorough understanding of the adverse effect profiles of prescribed agents and the risk factors predisposing to their development
Misinterpretation of drug-induced adverse effects as disease symptoms without consideration of ongoing pharmacotherapy	When new symptoms occur in a patient receiving pharmacotherapy, the primary consideration should be whether they are attributable to the treatment itself. If confirmed, appropriate modifications of the pharmacotherapy are required
Uncritical use of electronic tools for assessing the risk of drug–drug interactions in polypharmacotherapy	Most drug–interaction prediction tools fail to indicate the dosage thresholds at which interactions become clinically relevant, and only a limited number report interactions arising from the additive adverse effects of concomitantly used medications
Cumulative adverse effects of drugs in polypharmacotherapy as a source of complications in patients with multimorbidity	The additive effects of adverse reactions represent one of the most frequent forms of drug interactions in clinical practice. The symptoms that emerge through this mechanism frequently initiate a prescribing cascade.

**Table 3:** Clinically important prescribing cascades.

Initial Trigger (Drug/Situation)	Clinical Problem Observed	Typical Prescribing Cascade	Clinically Recommended Alternative Approach
Calcium channel blocker therapy	Peripheral edema	Addition of diuretic	Dose reduction or switching to another antihypertensive class
Diuretic therapy	Urinary urgency/incontinence	Introduction of overactive bladder medication	Reassessment of diuretic type, dose or indication
Antipsychotic treatment	Extrapyramidal symptoms	Use of antiparkinsonian agents	Reduction in dose or switch to atypical antipsychotic
Benzodiazepine therapy	Cognitive impairment	Introduction of cognitive enhancers (e.g., cholinesterase inhibitors)	Gradual benzodiazepine withdrawal
Benzodiazepine withdrawal	Agitation/irritability	Addition of antipsychotic	Slower tapering, non-pharmacological interventions
SSRI/SNRI treatment	Insomnia	Prescription of sedative/hypnotic drugs	Adjustment of administration time or switch of antidepressant
NSAID therapy	Increase in blood pressure	Addition of antihypertensive medication	Reassessment of NSAID indication; use of alternative analgesic
Urinary anticholinergics	Cognitive impairment	Introduction of cholinesterase inhibitor or memantine	Deprescribing of anticholinergics and reassessment of indications
Alpha-1 receptor blocker	Orthostatic hypotension, dizziness	Use of vestibular sedatives	Dose modification or change in antihypertensive strategy

**Table 4:** Other prescribing cascades.

Physiological System/Drug Category	Type of Adverse Effect	Example of Prescribing Cascade (Drug A → Clinical Effect)	Typical Drug B Introduced
Cardiovascular therapies	Hemodynamic/vascular reactions	ACE inhibitor → cough	Cough remedies
Cardiovascular therapies	Orthostatic intolerance	Antihypertensive → dizziness/orthostatic hypotension	Antiemetic
Cardiovascular therapies	Blood pressure elevation	Drug-induced hypertension → increased BP	Antihypertensive drugs
Cardiovascular therapies	Mood/sexual functioning	Lipophilic β-blocker → depression or erectile dysfunction	Antidepressant/PDE-5 inhibitor
Cardiovascular therapies	Fluid retention	Calcium channel blocker/gabapentin/pregabalin → peripheral edema	Diuretic
Cardiovascular therapies	Gastrointestinal motility	Calcium channel blocker → constipation	Laxative
Cardiovascular therapies	Metabolic imbalance	Diuretic → hyperuricemia/gout	Anti-gout therapy
Cardiovascular therapies	Urinary symptoms	Diuretic → urinary urgency/incontinence	Overactive bladder medication
Cardiovascular therapies	Muscle complaints	Statin → myalgia/myositis	Pain reliever/mineral supplement/quinine sulfate
Cardiovascular therapies	Sleep disturbance	Statin → insomnia	Hypnotic/sleep aid
Cardiovascular therapies	Rhythm/BP changes	Digoxin → nausea; Midodrine → hypertension	Antiemetic/antihypertensive
Central nervous system agents	Dermatological	Anticonvulsant → rash	Topical corticosteroid
Central nervous system agents	GI symptoms	Anticonvulsant → nausea	Antiemetic
Central nervous system agents	Motor symptoms	Antipsychotic → EPS/akathisia/tremor	Beta-blocker/antiparkinsonian/anti-tremor antimuscarinic/sedative
Central nervous system agents	Cardiovascular effects	Antipsychotic → arrhythmia	Antiarrhythmic
Central nervous system agents	Metabolic	Antipsychotic → hyperglycemia	Antihyperglycemic
Central nervous system agents	Cognitive	Benzodiazepine → cognitive impairment	Cholinesterase inhibitor
Central nervous system agents	Urinary	Cholinesterase inhibitor → urinary incontinence	Overactive bladder medication
Central nervous system agents	Sleep disturbance	Cholinesterase inhibitor → insomnia	Sleep agent
Central nervous system agents	GI irritation	Cholinesterase inhibitor → nausea, diarrhea, GI upset	Antiemetic, antidiarrheal, bismuth
Central nervous system agents	ENT symptoms	Cholinesterase inhibitor → rhinorrhea	Antihistamine
Central nervous system agents	Psychosis	Dopaminergic agents → hallucinations	Antipsychotic
Central nervous system agents	Tremor/BP elevation	Venlafaxine → tremor or hypertension	Benzodiazepine/antihypertensive
Central nervous system agents	Urinary	SSRI/SNRI → urinary incontinence	Overactive bladder medication
Central nervous system agents	Cognitive/GI	Tricyclic antidepressant → cognitive impairment, constipation, urinary issues	Cholinesterase inhibitor/laxative/overactive bladder medication
Endocrine therapies	Musculoskeletal	DPP-4 inhibitor → joint pain	NSAID
Endocrine therapies	Infections	SGLT-2 inhibitor → mycotic genital infections	Antifungal
Endocrine therapies	GI symptoms	Metformin → diarrhea	Antidiarrheal
Endocrine therapies	Fluid retention	Pioglitazone/rosiglitazone → edema	Diuretic
Endocrine therapies	Heart failure	Rosiglitazone → HF worsening	Diuretic
Gastrointestinal system drugs	Urinary	Anticholinergic antiemetic → urinary retention	Alpha-1 blocker
Gastrointestinal system drugs	Motor symptoms	Antidopaminergic antiemetic → EPS	Antiparkinsonian agent
Gastrointestinal system drugs	GI irritation	Laxative → diarrhea	Antidiarrheal
Gastrointestinal system drugs	Metabolic/bone	PPI → osteoporosis, fractures, vitamin deficiency	Vitamin/mineral supplementation
Musculoskeletal system drugs	GI toxicity	Bisphosphonate or NSAID → gastritis/ulcer/bleeding	Gastroprotective therapy
Musculoskeletal system drugs	Nausea	NSAID → nausea	Antiemetic
Musculoskeletal system drugs	BP changes	NSAID → hypertension	Antihypertensive
Musculoskeletal system drugs	Cardiac	NSAID → HF worsening	Heart failure medication
Musculoskeletal system drugs	Mood	Opioid → depression	Antidepressant
Urogenital system drugs	Orthostatic	Alpha-1 receptor blocker → dizziness/orthostatic hypotension	Vestibular suppressant
Urogenital system drugs	Dry mouth	Urinary anticholinergic → xerostomia	Saliva substitute
Miscellaneous therapies	Neurological	Carbapenem → seizures	Anticonvulsant
Miscellaneous therapies	Sleep changes	Corticosteroid → insomnia	Hypnotic
Miscellaneous therapies	Psychiatric	Corticosteroid → psychosis	Antipsychotic
Miscellaneous therapies	BP elevation	Corticosteroid or fludrocortisone → hypertension	Antihypertensive
Miscellaneous therapies	Dermatologic/GI	Acitretin → genital candidiasis	Antifungal
Miscellaneous therapies	Rhythm disturbance	Erythromycin → arrhythmia	Antiarrhythmic
Miscellaneous therapies	GI motility	Iron supplement → constipation	Laxative

**Table 5:** Prescribing cascade assessment tool.

Existence of ADR, either expected or unknown:	
Doubtful	0
Yes	1
Yes, but misunderstood	2
Action followed against the ADR:	
Treatment discontinuation	0
Continued with dose reduction	1
Continued unchanged or with another drug of the same group	2
Existence of a second drug treatment for the ADR:	
No	0
Yes	1
Overall result of this new treatment:	
Patient improves	0
Patient worsens or remains unchanged	1
A new ADR appears	2
The new ADR requires a third drug treatment	3

**Table 6:** Prescribing cascade benefit assessment tool.

Does/did the precipitating drug cause or pose a risk for a clinically relevant adverse drug reaction?
Is the precipitating drug still indicated?
Can a treatment adjustment of the precipitating drug prevent adverse drug reactions?
Can switching the precipitating drug prevent adverse drug reactions?
Can the second drug have a beneficial effect on adverse drug reactions?
Is the benefit–risk balance of the prescribing cascade positive?

**Table 7:** How to Prevent Prescribing Cascade—Table of Good Practice.

Area	Principle/Clinical Practice
Oligopharmacotherapy	Use only absolutely essential medications.
Zero tolerance for unnecessary drugs	Eliminate therapies without clinical indications.
Contextual pharmacotherapy	Take into account comorbidities and the individual situation of the patient.
Patient assessment	Before introducing a new drug, evaluate the entire pharmacotherapy, risk of interactions, and potential adverse effects.
Deprescribing	Regularly discontinue unnecessary or harmful medications.
Adverse drug reactions	First, consider discontinuing dietary supplements; Limit unnecessary self-medication; Respond immediately to new symptoms.
Informed patient	Educate the patient: treatment goals, risks of polypharmacy, and the need to report adverse effects.

## Data Availability

No new data were created in this study.

## Abbreviations

The following abbreviations are used in this manuscript:

AKI	Acute Kidney Injury
CKD	Chronic Kidney Disease
ACEI	Angiotensin Converting Enzyme Inhibitors
OTC	Over The Counter
EBM	Evidence-Based Medicine
PIPC	Potentially Inappropriate Prescribing Cascades
GERD	Gastroesophageal Reflux Disease
DDP-4	Dipeptidyl Peptidase 4
SGLT-2	Sodium Glucose Cotransporter 2
NSAID	Nonsteroidal Anti-Inflammatory Drug
ADR	Adverse Drug Reaction
